# Supplementary figures and images for: How peak knee loads are affected by changing the mass of lower-limb body segments during walking
Source: PLoS Comput Biol. 2025 Sep 24;21(9):e1012833. doi: 10.1371/journal.pcbi.1012833 (PMC12483208; doi:10.1371/journal.pcbi.1012833)

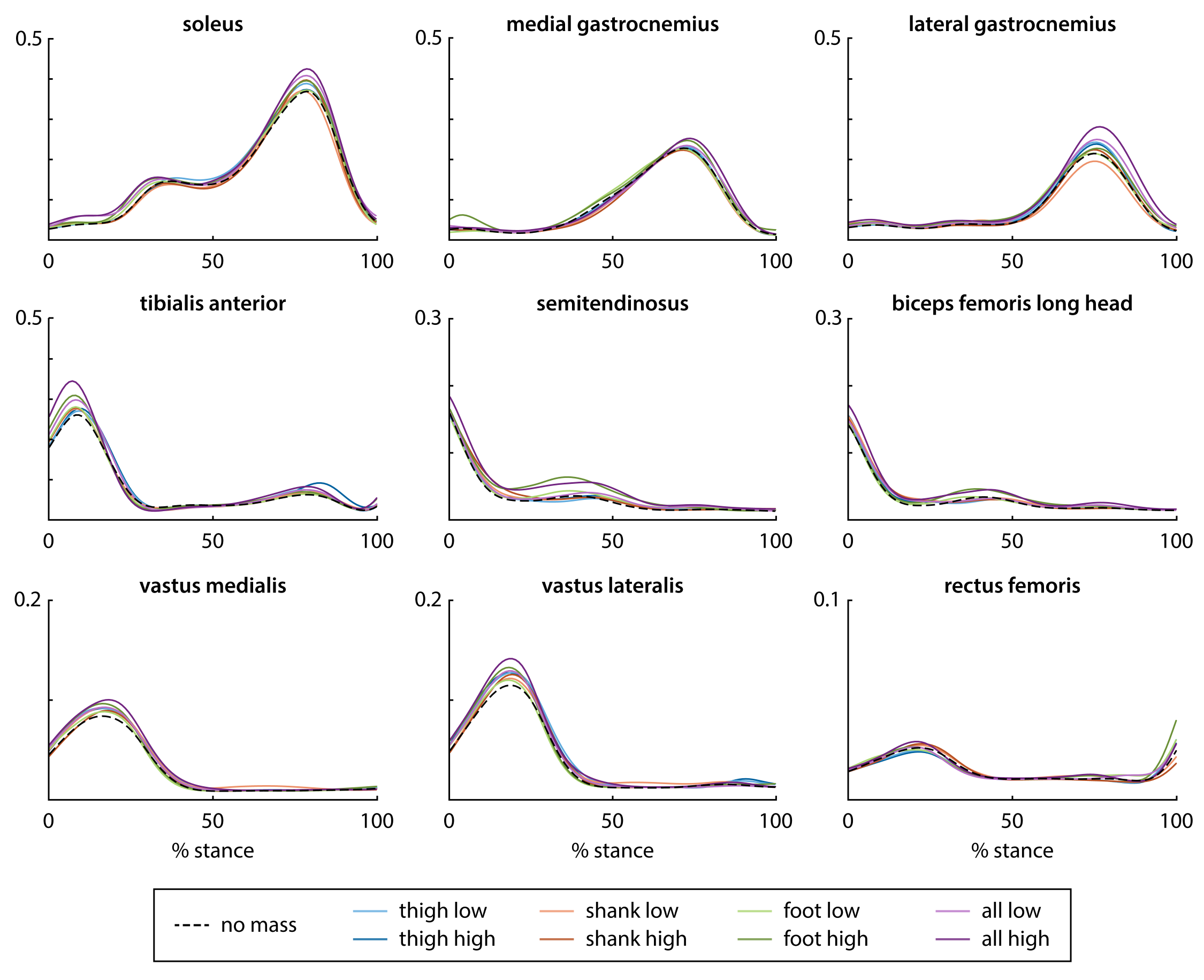

Supplement: S1 Fig — EMG waveforms during stance are shown for each loading condition, averaged across participants. EMG data were collected from 9 lower-limb muscles on the right leg. Raw signals were high-pass filtered at 20 Hz, full wave rectified, then low-pass filtered at 6 Hz. Filtered signals were normalized to the maximum seen across maximum voluntary contraction (MVC) and gait trials, then further scaled by during the Calibration stage of EMG-informed simulation. For each muscle, the loaded conditions are shown by color and the unloaded (no mass) condition is shown as a dashed black line. (TIF) [file pcbi.1012833.s001.tif]

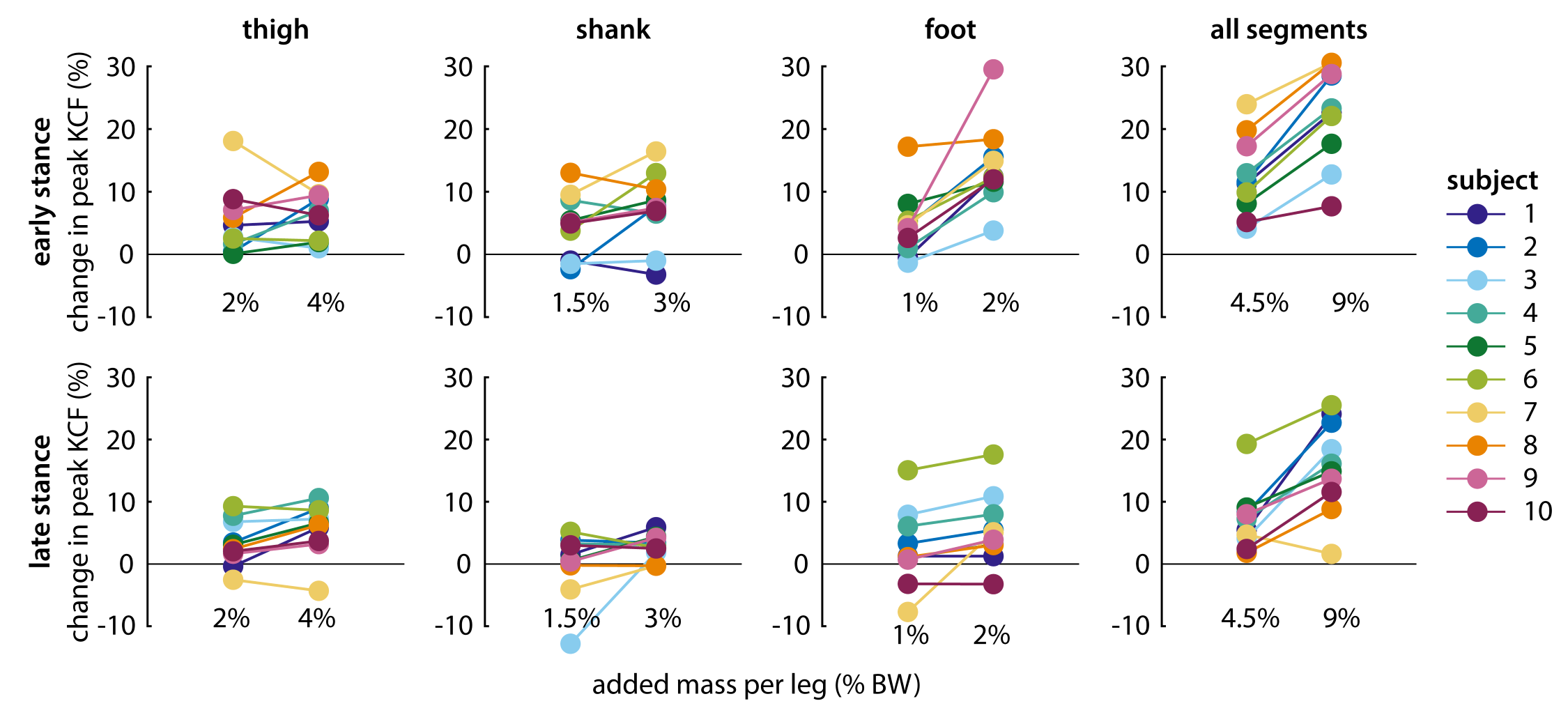

Supplement: S2 Fig — The relationship between added segment mass as a percentage of body weight (% BW) and percent change in peak knee contact force (KCF) is shown for each subject, represented by a different color. Each column indicates a different limb segment loading condition (thigh, shank, foot, all segments). The top row shows changes in early-stance peak KCF, and the bottom row shows changes in late-stance peak KCF. (TIF) [file pcbi.1012833.s002.tif]
